# A Model Hospital

**Published:** 1873-07

**Authors:** 


### EDITORIAL, ETC.

*A Model Hospital.*—Mr. C. C. Fulton, of Baltimore, now in Europe, sends the following description of the General Hospital of Vienna, which, in view of the fact that the Johns Hopkins' Hospital, soon to be erected in Baltimore, will be the largest and most complete in America, will prove interesting to our readers, as they will understand by this account of the Vienna institution what a magnificent hospital our city will in a short time possess, through the liberality of one of her most prominent citizens:

"This building was originally built for and used as an almshouse, under the reign of Leopold I., finished in 1769, and turned into a hospital during the reign of Joseph II., in 1784, who paid the cost of the alteration out of his private means. It is the largest hospital in Europe, and at that time already had room for two thousand beds. The entire building is of brick, two stories high. In 1832 the obstetrical wards were added, which is three stories high, and cost 496,831 florins. In 1862 the new pathological institute was finished, in which the post mortems are made. In this building all the clinical lectures are held, and in a few years it is expected that it will be entirely turned over to the University.

At present clinical instruction is given by 20 professors, 25 private docents, as they are called here, and 16 assistants. All persons (except children under five years of age, for whom there is a children's hospital separate from the above,) are admitted.

The grounds have a frontage on three streets. They consist of a series of buildings, divided by thirteen courts, with fountains in the centre, and cover a space of 26,718 square degrees. The buildings alone cover a space of 7,350 square degrees. Both the old building and the addition are built in the shape of parallelograms.

The smallest ward contains 14,495 cubic feet, the largest

49,187 cubic feet. There are 143 wards in the building, capable of accommodating 2,018 patients, for whom there are 220 nurses, mostly females, or about one to every ten patients.

For the purposes of clinical instruction, the following number of beds are at the disposal of the Medical Faculty, exclusive of clinical wards: 2 medical departments, clinical wards 50 beds; 1 mental disease department, clinical wards 50 beds; 2 surgical disease departments, clinical wards 50 beds; 2 eye disease departments, clinical wards 30 beds; 2 syphilis disease departments, clinical wards 52 beds; 1 skin disease department, clinical wards 47 beds; 1 throat disease department, clinical wards 18 beds; 2 ear disease departments, clinical wards 19 beds. All the rest of the cases of the institution are treated by a number of primararyte, as they are called, but who give no clinical instruction.

In the year 1871, 21,147 patients were admitted into the hospital, and 2,932 died. In connection with the regular clinics there is also an out-door system of prescribing, thousands being treated in this way annually.

The pathological-anatomical college, a beautiful building, two stories high, adjoining the Hospital, contains on the first floor rooms for post-mortem purposes. It will be necessary to premise that all persons dying in the Hospital before their bodies are delivered to their friends must be subjected to a post-mortem examination. If the friends are not able to pay the expenses of a funeral, then the body goes to the dissecting room during the lecture season (ten months in the year,) and is afterwards buried at the expense of the State. These post-mortem examinations have produced a "Rokitansky, a Virchow, and a Rindfleisch." The corpse, two hours after death, is brought into this building, and into a large room filled with beds and blankets, in one of which it is placed and covered. On the wall alongside of each bed is an alarm clock, with a string which is attached to the wrist of the body. The least movement that is made sets the bell in motion, and alongside the room is another in which there are always two men with restoratives at hand in case they should ever be needed. In the afternoon the bodies are examined by the assistants of the Professor of Pathological Anatomy, and after he [who, by the way, is sworn for this purpose] has declared them dead, they are undressed and placed in another room on iron stretchers. The next morning at 8 o'clock the post-mortem is made, and in the afternoon the body is turned over to the friends.

On this floor are three separate rooms for post-mortems, one for the judicial post-mortems made by Prof. Rokitansy himself, who is the sworn officer of the Government for this purpose. All cases of suspicious death, suicides, &c., in the city are sent to the Pathological building, and the post-mortem examinations are made in this room by the above mentioned Professor. Then there is the clinical post-mortem room, where the bodies are examined of those who have died in the clinical wards, generally in the presence of the Professor who treated them, and his students. In the last room is held the post-mortem examinations for the other departments of the Hospital.

Beside these rooms on the first floor are the working rooms of the Professors of Surgery and Medicine, and in the second story are the chemico-pathological rooms, rooms of the Professor of Experimental Pathology, lecture room for the pathological anatomy, and last, but most important, the pathological museum of Prof. Rokitansy, the finest in the world, containing 5,000 of the rarest specimens.

The number of physicians attached to the Hospital is eleven professors, twelve department physicians, who have charge of divisions not at all in connection with the professors, sixteen assistants to the professors, and forty assistants to the department physicians—with the Pathological Anatomy Professor and assistants makes a sum total of eighty-fcur physicians. There are also four priests who reside in the institution.

Every patient, as soon as he is admitted into the Hospital, receives the regular Hospital dress, and his own clothes, after being numbered, are locked up in a separate building by an officer appointed for this purpose. The cost of treatment is about 5 florins per week. If the patient is poor, it is charged to the village or city where he resides ; if a servant or apprentice, it is charged to the employer.

In the Eye Departments there are now two professors, and in the hall there will be three full clinics on this branch in operation. Professor Von Ault, who holds the highest position in Germany, has besides the regular clinic, the out-door patients daily from 11 to 12, the cases annually treated in this way being over 5,000. The assistants give private courses on the various branches of this specialty ; altogether about ten of these courses are given, each lasting about six weeks, and the same rule holds good in the other branches. For opportunities for studying, this Hospital stands unequalled.

In the Obstetrical Department, one of which is for students and one for midwifes, there are annually about eight to nine thousand births, of this number from two to three hundred are legitimate children—comment is unnecessary.

There were upwards of fifty American physicians here the past winter, the most of them from New York and Massachusetts, a number of them being professors of some of our medical schools."

